# Do early life cognitive ability and self-regulation skills explain socio-economic inequalities in academic achievement? An effect decomposition analysis in UK and Australian cohorts

**DOI:** 10.1016/j.socscimed.2016.07.016

**Published:** 2016-09

**Authors:** Anna Pearce, Alyssa C.P. Sawyer, Catherine R. Chittleborough, Murthy N. Mittinty, Catherine Law, John W. Lynch

**Affiliations:** a School of Population Health, University of Adelaide, Adelaide, Australia; b Population, Policy and Practice, UCL Institute of Child Health, University College London, London, United Kingdom; c School of Social and Community Medicine, University of Bristol, United Kingdom

**Keywords:** Socio-economic inequalities, Early childhood, Early intervention, Academic achievement, UK millennium cohort study, Longitudinal Study of Australian Children, Avon longitudinal study of parents and their children

## Abstract

Socio-economic inequalities in academic achievement emerge early in life and are observed across the globe. Cognitive ability and “non-cognitive” attributes (such as self-regulation) are the focus of many early years’ interventions. Despite this, little research has compared the contributions of early cognitive and self-regulation abilities as separate pathways to inequalities in academic achievement. We examined this in two nationally representative cohorts in the UK (Millennium Cohort Study, n = 11,168; 61% original cohort) and Australia (LSAC, *n* = 3028; 59% original cohort).

An effect decomposition method was used to examine the pathways from socio-economic disadvantage (in infancy) to two academic outcomes: ‘low’ maths and literacy scores (based on bottom quintile) at age 7–9 years. Risk ratios (RRs, and bootstrap 95% confidence intervals) were estimated with binary regression for each pathway of interest: the ‘direct effect’ of socio-economic disadvantage on academic achievement (not acting through self-regulation and cognitive ability in early childhood), and the ‘indirect effects’ of socio-economic disadvantage acting via self-regulation and cognitive ability (separately). Analyses were adjusted for baseline and intermediate confounding.

Children from less advantaged families were up to twice as likely to be in the lowest quintile of maths and literacy scores. Around two-thirds of this elevated risk was ‘direct’ and the majority of the remainder was mediated by early cognitive ability and not self-regulation. For example in LSAC: the RR for the direct pathway from socio-economic disadvantage to poor maths scores was 1.46 (95% CI: 1.17–1.79). The indirect effect of socio-economic disadvantage through cognitive ability (RR = 1.13 [1.06–1.22]) was larger than the indirect effect through self-regulation (1.05 [1.01–1.11]). Similar patterns were observed for both outcomes and in both cohorts.

Policies to alleviate social inequality (e.g. child poverty reduction) remain important for closing the academic achievement gap. Early interventions to improve cognitive ability (rather than self-regulation) also hold potential for reducing inequalities in children's academic outcomes.

## Highlights

• We decomposed pathways from socio-economic disadvantage to mid-childhood academic ability.
• 2/3 effect of socio-economic disadvantage was direct (not through cognitive ability *or* self-regulation).
• The indirect effect was mainly through cognitive ability.
• Early interventions to improve cognitive ability hold potential.
• But policies to alleviate social inequality remain essential for inequality reduction.

## 1 Introduction

Educational qualifications and trajectories of employment, income and health across the life course are all importantly influenced by academic achievement in childhood (Galobardes et al., 2008, Harper et al., 2011). There are large socio-economic inequalities in academic achievement throughout childhood (Brinkman et al., 2012, Sirin, 2005), and these help drive the emergence of health inequalities (Lynch and Davey Smith, 2005). In acknowledgement of the benefits to giving every child a strong start in life and the subsequent contributions to the economic productivity of society (Allen, 2011, Organization for Economic Cooperation and Development, 2011), the focus of government and non-government organizations in many countries has turned to improving overall levels and socio-economic gaps in academic achievement in early childhood (Douglas et al., 2014, HM Government, 2011, Organization for Economic Cooperation and Development, 2011, The Equity and Excellence Commission, 2013).

While cognitive ability is a widely recognised determinant of academic achievement, there is increasing interest in the role of “non-cognitive” characteristics (F Cunha and Heckman, 2007, Heckman et al., 2006, Kautz et al., 2014). Though the term “non-cognitive” has not been consistently defined or measured, the idea of non-cognitive skills encapsulates personality characteristics and social behaviours that can maximise life opportunities (Borghans et al., 2008). In young children an important component of non-cognitive abilities is self-regulation (Barkley, 2011) which refers to the control of attention, emotion and behaviour (Blair and Diamond, 2008). Some research has suggested that early “non-cognitive” skills like self-regulation may be as important (if not more important) than cognitive ability for future outcomes like labour market success, both directly and by supporting later cognitive ability (Flavio Cunha and Heckman, 2008).

Self-regulation is integral to cognitive ability in childhood, through supporting engagement in and persistence with learning tasks (Blair and Diamond, 2008). Cognitive ability and self-regulation have both been linked to better academic achievement (Blair and Diamond, 2008, Oberle et al., 2014, Sawyer et al., 2015) and are generally lower among socially disadvantaged children (C. R. Chittleborough et al., 2014, Dearden et al., 2011, Evans and Rosenbaum, 2008, Feinstein, 2003, Sektnan et al., 2010). Observational studies indicate that self-regulation (Dilworth-Bart, 2012, Evans and Rosenbaum, 2008, Sektnan et al., 2010) and cognitive ability (C. R. Chittleborough et al., 2014) may mediate the association between socio-economic disadvantage (SED) and academic achievement (although none explicitly compared the mediating roles of both). It is therefore plausible that intervening on these components of child development (Bierman et al., 2008, Raver et al., 2011) may reduce socio-economic inequality in academic achievement. Interventions targeting cognitive ability and/or self-regulation in the United States have been shown to improve school readiness and early academic achievement (Kautz et al., 2014), including in disadvantaged families (Bierman et al., 2008, Raver et al., 2011), although effects may fade with time (Burger, 2010, U.S. Department of Health and Human Services and Administration for Children and Families, 2010). A comparison of cognitive and self-regulation skills, as two related mechanisms that can be targeted by interventions, would inform the design of early childhood programs to reduce socioeconomic gaps in academic achievement.

**Fig. 1:**
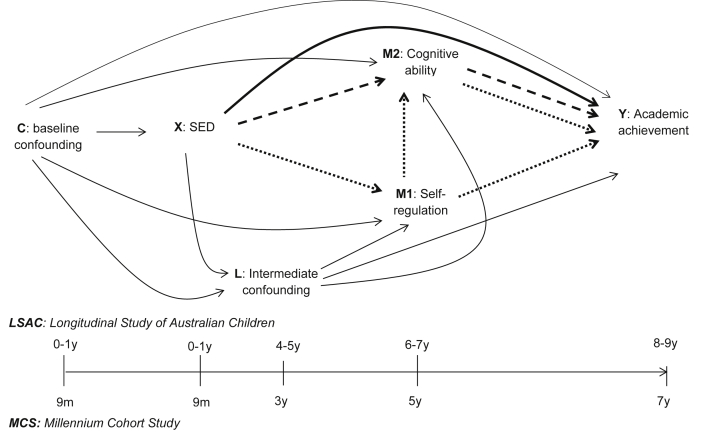
Directed Acyclic graph (DAG) of the direct pathway (shown in bold) from socio-economic disadvantage (SED) (X) to academic achievement (Y), the indirect pathways via self-regulation (M1, shown in dots) and cognitive ability (M2, shown in dashes), and baseline (C) and intermediate confounding (L).

Our goal was to decompose the pathways from SED at birth (represented by low maternal education) to children's academic achievement in mid-childhood that were via early-life self-regulation (task attentiveness and persistence) and cognitive ability (verbal and non-verbal skills). Fig. 1 shows the direct pathway from SED to the child academic achievement (in bold), the indirect pathway via cognitive ability (in dashes), and the indirect pathway via self-regulation (including via cognitive ability in dots). We conducted comparative analyses throughout early- to mid-childhood using data from contemporary, nationally representative cohorts from Australia (the Longitudinal Study of Australian Children, LSAC(Australian Institute of Family Studies, 2014)) and the United Kingdom (UK) (the Millennium Cohort Study, MCS(Connelly and Platt, 2014)). As a sensitivity analysis to measurement error in the self-regulation measures, which were based on maternal report in MCS and LSAC (see Methods), we examined these associations in a third cohort - the Avon Longitudinal Study of Parents and Children, ALSPAC(Boyd et al., 2012, Fraser et al., 2013), which collected an objective measure of executive function, a measure of self-regulation in young people.

## 2 Methods

### 2.1 Participants

#### 2.1.1 Longitudinal Study of Australian Children

The LSAC is a nationally representative prospective study of two cohorts of children, recruited 2003–2004. The methodology has been previously described (Soloff et al., 2005). We used data on 5107 infants (64% of those invited to take part) from the ‘b-cohort’, who were first contacted at 0–1 year.

#### 2.1.2 The Millennium Cohort Study

The MCS is a longitudinal study of children born in the UK, 2000–2002. Information on the survey design has been described elsewhere (Hansen, 2010). The first contact with the cohort child was carried out at around age 9 months for 18,818 infants (91% of the 20,646 of the target sample). Data were downloaded from the UK Data Service, University of Essex and University of Manchester, in April 2014.

In both cohorts, interviews were carried out with trained interviewers in the home, with the primary caregiver (usually the mother) and her partner (if relevant); postal questionnaires were also sent to the children's teachers once they reached school age.

### 2.2 Measures

The counterfactual analytical method used to decompose the mediating pathways of interest (Vanderweele et al., 2014) (see Analysis) favours use of binary exposure, mediator and intermediate confounding variables, because the availability of just one counterfactual state aids interpretability of results. All measures are described in detail in Table 1 and summarised below, including cut-offs for dichotomisation (where relevant).

#### 2.2.1 Exposure: socio-economic disadvantage

Mothers’ highest educational qualifications (when the cohort child was an infant) were used as indicators of SED. Low education was defined by educational targets set by the Australian (completion of Year 12(The Commonwealth of Australia and the States and Territories, 2009)) and UK (General Certificate in Secondary Education (GCSE), grades A*-C(HM Government, 2011)) governments.

#### 2.2.2 Outcome: low academic achievement

We analysed two separate measures of academic achievement: maths and literacy scores derived from teacher assessment in LSAC and by tests completed by the MCS children during the interview. ’Low’ academic achievement was defined as being in the lowest quintile of scores.

#### 2.2.3 Mediator 1: low self-regulation

We used a number of items representing a component of self-regulation known to influence academic achievement - task attentiveness and persistence (Sawyer et al., 2015) (see Table 1). Responses to the items were summed to create self-regulation scores (Table 1); children in the lowest quintile were defined as having ‘low’ self-regulation.

#### 2.2.4 Mediator 2: low cognitive ability

Cognitive ability was defined as the non-verbal and verbal abilities of the child (Table 1). Non-verbal abilities were assessed with the Matrix Reasoning subtest in LSAC and pattern construction in the MCS. Verbal abilities were assessed using a test of receptive vocabulary. Verbal and non-verbal scores were standardised using T-scores and then combined (Connelly, 2013). The lowest quintile was used to represent ‘low’ cognitive ability.

#### 2.2.5 Baseline confounding

Baseline confounders were young maternal age (<20 years) at first live birth and language spoken in the home (English/other). MCS analyses were repeated adjusting for ethnicity in place of language and the results were unchanged (ethnicity was not collected for non-indigenous children in LSAC).

#### 2.2.6 Intermediate confounding

The following were considered to confound the mediator/outcome association and were also associated with the exposure: alcohol consumption and smoking in pregnancy, and at ages 3–5: lone parenthood status, housing tenure, household income, household unemployment, maternal psychological distress, parenting style and formal childcare use.

**Fig. 2:**
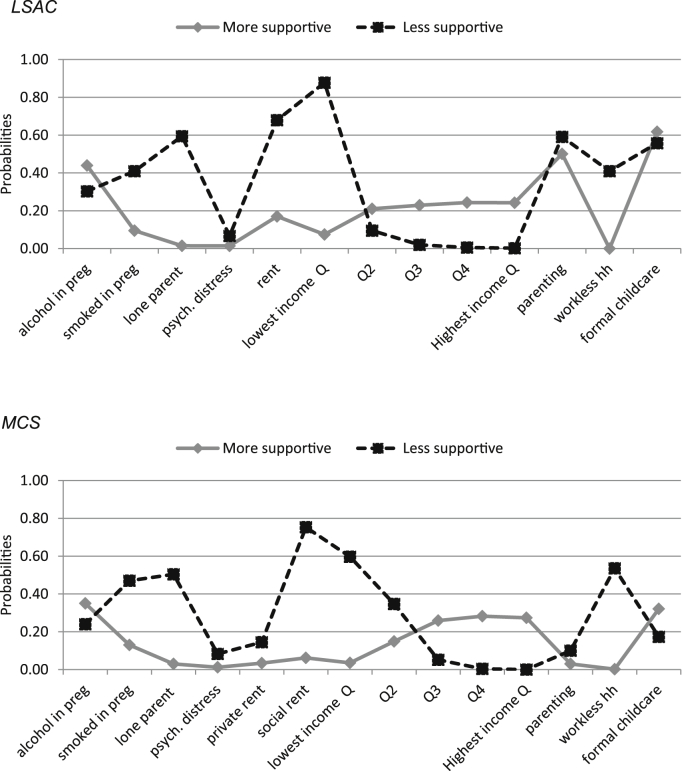
Characteristics of the latent variable used to represent intermediate confounding (more and less supportive home and parenting environments).

Latent class analysis (LCA) was used to create a summary measure of confounding characteristics (referred to hereafter as the ‘Early home and parenting environment’). A two class model offered a good fit in both cohorts (see Table A1, Appendix A), with good separation for all items except alcohol in pregnancy, maternal psychological distress, parenting style and formal childcare use. The resulting binary variable (representing the two classes) distinguished between less and more supportive environments (Fig. 2). The LCA was carried out in Stata 13.0 (StataCorp, College Station, TX) using a Stata plug-in for the SAS procedure PROC LCA (Lanza et al., 2007).

### 2.3 Analysis

We used a counterfactual method for decomposing two related mediating pathways (Vanderweele et al., 2014). In counterfactual methods, the observed data are used to estimate the potential outcome that would have been observed had exposed individuals been unexposed, and unexposed individuals been exposed (Rubin, 2005). Therefore estimates refer to average change in outcomes when individuals' observed exposure status is manipulated to the counterfactual (for example, if less advantaged families were made more advantaged). Some counterfactual methods allow the value of the mediator to react to the change in the exposure from its observed to its counterfactual state, enabling estimation of *natural* indirect and direct pathways (Lange et al., 2014, Vanderweele et al., 2014) (although issues of interpretation of natural direct and indirect ‘effects’ have been raised (Naimi et al., 2014)). Estimating natural direct and indirect pathways can be problematic when the mediator is subject to intermediate confounding (i.e. when a confounder of the mediator–outcome relationship is induced by the exposure) or when there are multiple, related mediating pathways (Vanderweele et al., 2014). VanderWeele, Vansteelandt and Robins demonstrate a series of analytical approaches that enable the estimation of direct and indirect pathways in the presence of intermediate confounding, or two related mediators (Vanderweele et al., 2014).

The first of VanderWeele, Vansteelandt and Robins' analytical approaches, referred to as ‘*Joint mediators*’, provides an effect estimate of the ‘direct’ pathway from exposure to outcome that is not acting via the two mediators (*M1* and *M2*, where *M1* is a cause of *M2*), and another for the joint indirect pathway through two related mediators (Vanderweele et al., 2014). This approach might therefore be used to examine the potential for a single intervention, which improves both self-regulation and cognitive ability, to reduce inequality in academic achievement. The direct pathway is given by the change in risk of the outcome when the value of the exposure is altered from its observed to its counterfactual value (while the mediators are held at their observed values). The joint indirect pathway is the difference in the risk of the outcome when both mediators are changed from their observed to their counterfactual values (had the exposure taken the opposite value), while the exposure is held at its observed value. A more detailed explanation and statistical notation are provided in Appendix B.

The second approach, ‘*Path specific effects*’, estimates the direct pathway in the same way, but in addition decomposes the joint indirect pathway into that through each mediator separately (Vanderweele et al., 2014). This approach is therefore appropriate for comparing an intervention designed to improve cognitive ability with an intervention to improve self-regulation (which could in turn influence cognitive ability). The direct pathway is estimated using approach 1. The indirect pathway through the main mediator of interest (*M2*) is given by the difference in risk of the outcome when *M2* is changed from its observed to its counterfactual value; the exposure is held at its observed value, while the second related mediator (*M1*) is held at its counterfactual value. The indirect pathway through *M1* is given by the difference in the risk of the outcome when *M1* is changed from its observed value to its counterfactual value; while the exposure is held at its observed value, and *M2* (which is caused by both the exposure and *M1*) is held at a *new* counterfactual value (under the observed exposure but counterfactual *M1*). See Appendix B for further detail.

The third approach, referred to as ‘*Intervention effects*’, aims to emulate a randomized intervention. It provide an effect estimate for just one mediating pathway, while adjusting for the second related mediator (or an intermediate confounder), within levels of the exposure, using inverse probability weights (*IPTW*s) (Vanderweele et al., 2014). The effect estimate of the direct pathway refers to the pathway from SED to academic ability that it not acting through the single mediator of interest (after adjustment for intermediate confounding). This approach is therefore suited to situations where there is just one mediating pathway of interest, which is likely to be biased by intermediate confounding. The indirect effect is given by the change in the risk of the outcome when the value of *M2* is estimated (adjusting for *M1*) within levels of the observed exposure and within levels of the counterfactual exposure. The direct effect is estimated by changing the exposure from its observed to its counterfactual value, while the value of *M2* is held at the value it would have taken if assigned (adjusting for *M1*) within levels of the counterfactual exposure. See Appendix B.

**Fig. 3:**
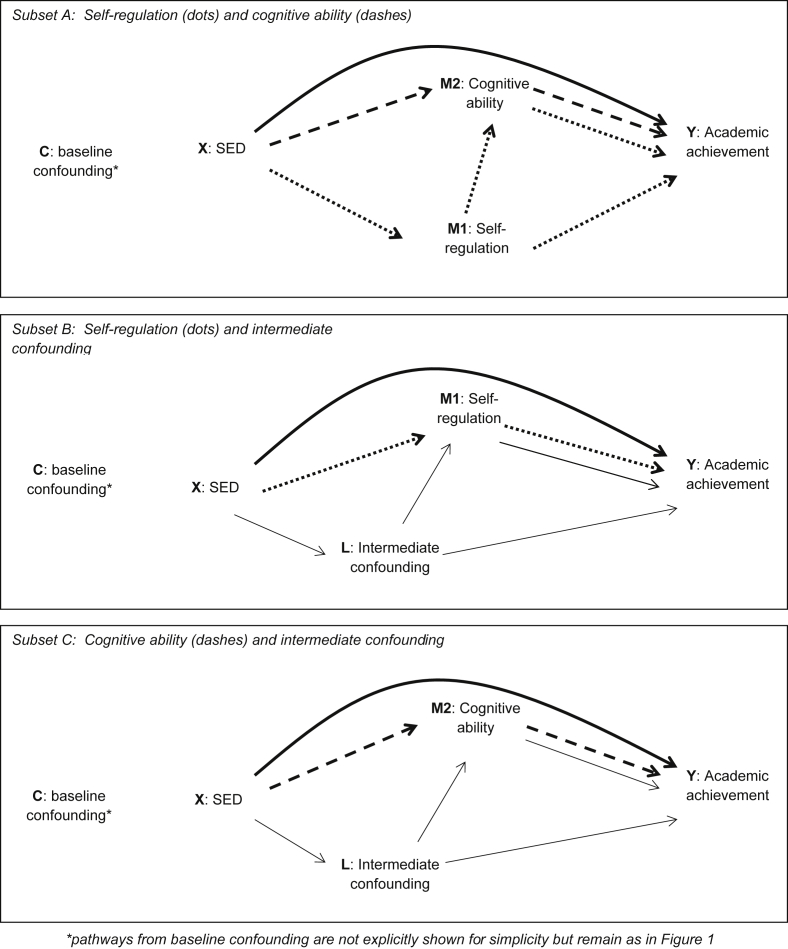
“Subsets” of the main Directed Acyclic Graph (Fig. 1) used to carry out analysis steps.

The directed acyclic graph (DAG, Fig. 1) demonstrates the main pathways of interest: the direct pathway from SED (*X*) to academic achievement (*Y*), and indirect pathways via the two related mediators: self-regulation (M1) and cognitive ability (*M2*). The DAG also includes intermediate confounding (*L*). Because none of the analytic approaches allow examination of two mediators *and* adjustment for an intermediate confounder in a single model (Vanderweele et al., 2014), we carried out a series of analyses in the following steps, each focussing on a different ‘subset’ of the DAG:

• *‘Step A: Effect decomposition via Self-regulation & Cognitive ability’* (Fig. 3a): in this step we focused on the two mediators of interest and disregarded intermediate confounding by *L.* Firstly, using the ‘Joint indirect effects’ approach, effect estimates for the direct pathway from SED to academic achievement (via neither of the mediators) and a joint indirect pathway via self-regulation (dotted line) *and* cognitive ability (dashed line) were estimated. This indirect pathway was then decomposed, using ‘Path specific effects’, to provide two separate effect estimates for the indirect pathway via cognitive ability, and the indirect via self-regulation (either directly, or via cognitive ability - because we hypothesized that the relationship between the mediators ran from self-regulation to cognitive ability).
• *‘Step B: Self-regulation & intermediate confounding’* (Fig. 3b): In Step B we estimated the indirect pathway from SED to academic achievement via self-regulation after adjusting for confounding by *L* (with IPTWs), using the ‘Intervention analogue’ approach. Cognitive ability was not included in this model.
• *‘Step C: Cognitive ability & intermediate confounding’* (Fig. 3c)*:* Here the ‘Intervention analogue’ approach was used to examine the degree to which the indirect pathway through cognitive ability was confounded by *L.* Self-regulation was not included in this model.

Findings from Steps A-C were then subjectively triangulated, in order to compare the mediating roles of self-regulation and cognitive ability (Step A) and the extent to which each of the indirect pathways might have been confounded (Steps B and C). Baseline confounders (*C*) were adjusted for in all analyses.

#### 2.3.1 Statistical modelling

Effect estimates for direct and indirect pathways from SED to maths and literacy scores (as separate outcomes) were estimated using binary regression, in form of the risk ratios (RRs, representing relative inequalities), and risk differences (RDs, representing absolute inequalities). 95% confidence intervals (CIs) were estimated using 5000 non-parametric bootstrap samples. Analyses were conducted in Stata/SE 13.0 (StataCorp, College Station, TX). Annotated Stata code is provided in Appendix B.

**Fig. 4:**
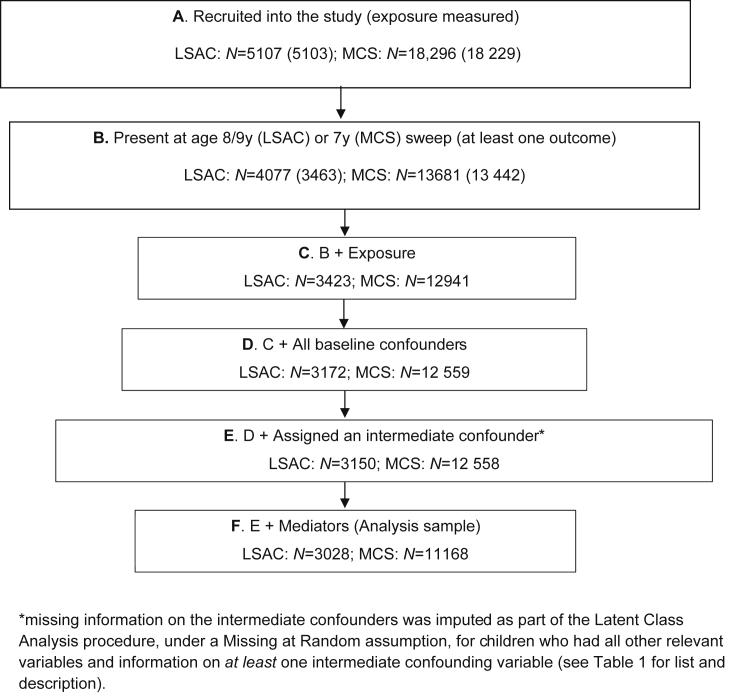
Flowchart of how analysis samples were obtained from original samples.

Given the complexity of the methods applied, we did not multiply impute the data and all analyses were carried out in a complete case sample. Fig. 4 shows how the analysis samples for the main models were obtained. Table A2 (Appendix C) compares the characteristics of response samples to complete case samples.

#### 2.3.2 Sensitivity analyses

It was only possible to adjust for one exposure-induced intermediate confounder (Vanderweele et al., 2014). Therefore several different variables representing the early home and parenting environment were combined in a two class latent variable. Although this measure provided a good fit in both cohorts, it is likely that the degree of intermediate confounding will be underestimated. We therefore repeated our analyses adjusting for individual confounding variables which were less well differentiated in the latent measure: maternal psychological distress, parenting style, formal childcare use.

School quality is an important determinant of academic achievement. In the case of LSAC children, it is also possible that school quality will have influenced self-regulation and cognitive skills (because these were captured at age 6–7). Therefore the indirect pathway from SED to academic outcomes via self-regulation and cognitive ability may have been overestimated, due to our inability to adjust for school quality. To address this we carried out a sensitivity analysis to unmeasured confounding in the joint indirect effect (VanderWeele and Chiba, 2014).

In LSAC and MCS, self-regulation was captured using a series of maternally reported questions about task attentiveness, whereas cognitive development was captured using tests. Because indirect pathways may be underestimated if a mediating variable is poorly measured (Blakely et al., 2013), we repeated our analyses in the UK ALSPAC(Boyd et al., 2012, Fraser et al., 2013), which included an objective measure of self-regulation in young people.

Finally, analyses were repeated using an alternative measure of SED (lowest household income quintile), alternative cut-offs for the self-regulation and cognitive ability measures (lowest two quintiles in place of the lowest quintile), and continuous maths and literacy scores in place of the binary measures.

## 3 Results

### 3.1 Descriptive statistics

#### 3.1.1 Socio-economic inequalities in the academic achievement (outcome) and cognitive ability and self-regulation (mediators)

Table 2 shows that, in both cohorts, the prevalence of low maths and literacy scores was almost twice as high in children from less advantaged backgrounds. For example the prevalence of poor maths scores was 30.9%, as compared to 16.2% in the more advantaged group (RR = 1.91 [1.59, 2.28]) in LSAC, and 30.6% compared to 16.4% (RR 1.87 [1.74, 2.01]) in MCS. Children from less advantaged backgrounds were also more likely to have low self-regulation and cognitive ability, although differences were greater for cognitive ability. For example, in LSAC the RRs for cognitive ability and self-regulation were 1.79 (1.50, 2.13) and 1.24 (1.03, 1.49).

#### 3.1.2 Academic achievement (outcome) according to self-regulation and cognitive ability (mediators)

As shown in Table 3, LSAC and MCS children with low self-regulation scores were around twice as likely to have low maths and literacy scores, compared to children who did not have low self-regulation. For example the prevalence of low maths scores was 28.2% of LSAC children in the lowest quintile of self-regulation scores, compared to 15.0% in those from all other quintiles. A stronger association with maths and literacy scores was observed for cognitive ability than self-regulation, particularly in the MCS where children with low cognitive ability were around three times as likely to have low maths scores (47.4% vs. 14.3%).

### 3.2 Decomposition of direct and indirect pathways from socio-economic disadvantage to academic achievement

Table 4 presents the decomposition of relative inequalities (using RRs) in Maths and Literacy scores. Absolute inequalities (represented by RDs) are decomposed in Table 5. Section A of the tables contain effect estimates for the direct pathway from SED to academic scores, the joint indirect pathway via self-regulation and cognitive ability, and the decomposed indirect pathways via self-regulation and cognitive ability separately. Sections B and C present the effect estimates for the indirect pathways, after adjustment for intermediate confounding (by *L*).

#### 3.2.1 Total ‘effects’ (direct and indirect pathways combined)

Addition of the RRs from the direct and joint indirect pathways (Section A, Table 4) indicated that, in total, children who were living in less advantaged families were around two thirds more likely to have low maths scores (RRs were around 1.65). For poor literacy scores, the combined RRs ranged from 1.7 to 1.9 (Section A, Table 4). In absolute terms (see Table 5), the total prevalence difference between children from more and less advantaged families was 12–13% for maths and 13–15% for literacy. The decomposition of these total ‘effects’ are now discussed.

#### 3.2.2 Direct and indirect pathways via self-regulation and cognitive ability

In both cohorts, the direct pathway from SED to low maths scores accounted for around two thirds of the total ‘effect’, meaning that just one third of the total ‘effect’ was acting through self-regulation and/or cognitive ability. When the joint indirect pathway was decomposed, the pathway via cognitive ability was considerably larger than the one via self-regulation. Similar patterns were observed for literacy. Using low maths scores (Table 4) in LSAC as an example: the RR for the direct pathway from SED to maths scores was 1.46 (1.17–1.79) and the joint indirect pathway via self-regulation *and* cognitive ability was 1.19 (1.10–1.32). The path specific analysis indicated that the majority of the joint indirect pathway was via cognitive ability (1.13 [1.06–1.22]) and not self-regulation (1.05 [1.01–1.11]). Decomposition of absolute inequality (risk differences) were similar: the direct pathway carried a RD of 7.51% (2.87, 12.59), with 3.00% (1.37, 5.10) and 1.12% (−0.13, 1.63) for the indirect pathways via cognitive ability and self-regulation respectively (Table 5).

### 3.3 Intermediate confounding on the indirect pathways through self-regulation and cognitive ability

Section B of Table 4 shows that the small indirect pathway from SED to academic achievement through self-regulation was not attenuated after adjustment for intermediate confounding. As can be seen in Section C of Table 4, the indirect pathway via cognitive ability increased very slightly, despite adjustment for intermediate confounding, because the part of the indirect pathway from self-regulation to cognitive ability was not excluded (as it was in Section A). Similar patterns were seen for absolute inequalities (Table 5).

### 3.4 Sensitivity analyses

We repeated the analyses in ALSPAC, which contains objective measures of executive function (a component of self-regulation in young people) and cognitive ability (Avon Longitudinal Study of Parents and Children). Because these measures were only collected in adolescence, findings are not directly comparable to the LSAC and MCS. However, this sensitivity analysis confirmed that the indirect pathway via cognitive ability was considerably larger than for self-regulation (Appendix D, Table A3).

A series of sensitivity analyses adjusted for individual intermediate confounding measures which were less well differentiated in the LCA (mother's psychological distress, parenting and formal childcare use) in separate models. Overall conclusions were unchanged.

A sensitivity analysis to unmeasured confounding by school characteristics (in LSAC only) indicated that the association between self-regulation and cognitive ability would have had to have been overestimated by 30% in order for the indirect pathway to have been completely removed. A more likely bias of 5% reduced the joint indirect pathway by a minimal amount. For maths scores the RR for the indirect pathway fell from 1.19 to 1.15 (and the direct effect increased from 1.46 to 1.49). For literacy scores the RR for the indirect pathway fell from 1.16 to 1.12 (and the direct effect increased from 1.51 to 1.54).

Similarly conclusions were unchanged when analyses were repeated with an alternative measure of SED (income), alternative cut-offs for the self-regulation and cognitive ability measures (capturing children in the lowest *two* quintiles), and continuous maths and literacy scores (data available on request).

## 4 Discussion

### 4.1 Summary of findings

We examined the potential for cognitive ability and self-regulation at the start of school to reduce inequalities in academic achievement at ages 7–9 in the UK and Australia. Children from less advantaged backgrounds (i.e. whose mothers left high school without Year 12 qualifications (Australia) or GCSEs grades A*-C (UK)) were around 1.6–1.9 times more likely to be in the lowest quintile of maths and literacy scores than those from more advantaged backgrounds. In terms of absolute inequalities, the prevalence of poor academic achievement in children from less advantaged backgrounds was 12%–15% higher than in those who were living in more advantaged families.

About two-thirds of the association between SED and children's academic abilities was direct (i.e. not mediated by self-regulation or cognitive ability). Decomposition of the indirect pathway showed that around 80–90% was through cognitive ability rather than self-regulation, in part reflecting the weaker association between self-regulation and both the exposure (maternal education) and the outcome (academic achievement). These findings were consistent when repeated with an alternative measure of SED (low income).

### 4.2 Methodological considerations

It was not possible to separately decompose two mediating pathways while also adjusting for intermediate confounding. However, we were able to account for intermediate confounding for one mediating pathway at a time. Intermediate confounding was captured using a binary latent variable representing a number of characteristics. A two class measure provided a parsimonious representation of the data, but it remains likely that the degree of confounding has been underestimated. However, sensitivity analyses adjusting for the characteristics which were least well differentiated in the latent measure indicated a similar level of confounding as seen in the main models. Additional sensitivity analyses (VanderWeele and Chiba, 2014) also implied that the conclusions are unlikely to be the artefact of unmeasured intermediate confounding.

In addition to the above limitations, which are specific to the analysis used, our findings are subject to the standard assumptions of sample representativeness, generalisability and measurement error. Around 70% of children who took part in the initial sweeps of LSAC and MCS had information on the exposure and outcome, and of these around 10% were missing baseline confounders or mediators (very few were missing intermediate confounding data because the latent class analysis was carried out under a missing at random assumption). However, findings were consistent for both outcomes and between cohorts. Additionally, conclusions were unchanged when analyses were repeated with an alternative measure of SED (low income), when using continuous maths and literacy scores in place of the binary outcomes, and when using an alternative cut-off in the mediating variables. There were differences in the measurement tools used in the MCS and LSAC which meant that results are not directly comparable. However we believe the consistency of findings between two different countries (Australia and UK), and in early (MCS, LSAC) and mid-late childhood (ALSPAC), indicate that these findings are generalisable to other high income settings. Finally, a sensitivity analysis in ALSPAC, which has objective measures of self-regulation, indicated that the smaller mediating pathway via self-regulation (compared to cognitive ability) was unlikely to be due to measurement error.

### 4.3 Concordance with previous research

Our findings are in agreement with the research of Cunha, Heckman and colleagues, which found that (in United States White males) cognitive ability was more important than “non-cognitive” skills for academic attainment upon leaving school (although it was less important than “non-cognitive” skills for labour market success) (Flavio Cunha and Heckman, 2008, Heckman et al., 2006). A number of studies examining self-regulation (Dilworth-Bart, 2012, Evans and Rosenbaum, 2008, Sektnan et al., 2010) or aspects of cognitive ability (C. R. Chittleborough et al., 2014) as mediators between SED and academic achievement in childhood indicate that both play a part. However, to our knowledge, ours is the first study to decompose and compare their contributions to socio-economic inequalities in childhood academic achievement.

### 4.4 Implications for equity interventions

Our results suggest that reducing social inequality (for example through increasing access to higher education in tomorrow's parents, or decreasing child poverty) remains an important strategy for narrowing inequalities in academic achievement and preventing the inter-generational transfer of social disadvantage. In the medium and shorter-term, interventions to support cognitive ability (rather than self-regulation skills) hold potential for reducing the socio-economic gap in academic achievement. Health, early care and education systems already reach almost the entire population and have a duty and a commitment to act now. Early cognitive ability is routinely monitored in Australia (Australian Government Department of Education and Training, 2015) and the UK(NHS England, 2014) and it is an integral focus of the national early years learning frameworks (Australian Government Department of Education and Employment and Workplace Relations for the Council of Australian Governments, 2009, Department for Education, 2012). The impact of these universal services on school readiness and academic achievement should be monitored into the future. Pro-equity progressive universal approaches are likely to be most successful for the improvement of academic achievement and inequality reduction (C. R. Chittleborough et al., 2014), because some families will require more support than others. However, identifying those who may benefit most from additional support remains a challenge (C. Chittleborough et al., 2011, Smithers et al., 2014).

## Figures and Tables

**Table 1:** Summary of variables.

Longitudinal Study of Australian Children (LSAC)		Millennium Cohort Study (MCS)	
**Socio-economic disadvantage (SED): Maternal education (0–1 years)**		**Socio-economic disadvantage (SED): Maternal education (9 months)**	
*Mothers' highest level of educational attainment*	• Low: Did not complete year 12^a^ • High: Completed year 12, certificate/diploma, degree	*Mothers' highest academic qualification*	• Low: GCSE^b^ grades D-G, or below • High: GCSE grades A*-C, A-Levels^c^, Diploma, Degree
**Academic achievement (8–9 years)**		**Academic achievement (7 years)**	
*Maths*	• Maths domain of Academic Rating Scale (ARS), teacher report (Australian Institute of Family Studies, 2011, Rothman, 2009) • Age standardised scores were divided into quintiles, with the bottom quintile representing ‘low achievement’	*Maths*	• Shortened version of the National Foundation for Education Research standard Progress in Maths test (Connelly, 2013) • Completed by the cohort child • Scores were divided into quintiles, with the bottom quintile representing ‘low achievement’ • Quintiles were assigned within each school year because scores were not age standardised (Connelly, 2013) (1% children were in ‘year 1’, 94% ‘year 2’, 5% ‘year 3’)
*Literacy*	• Literacy domain of the ARS, completed by the teacher (Australian Institute of Family Studies, 2011, Rothman, 2009). • Scores were standardised in a Rasch model, and divided into quintiles • ‘Low achievement’ = bottom quintile of scores	*Literacy*	• British Ability Scales II (BAS II) subtests for word reading, completed by the cohort child (Connelly, 2013) • Age standardised scores were divided into quintiles • ‘Low achievement’ = bottom quintile of scores
**Self-regulation (6–7 years)**		**Self-regulation (5 years)**	
*Task attentiveness and persistence*	• Previously created measure Sawyer et al., 2015 consisting of: • Five items from the Short Temperament Scale (When child starts a project… he/she works on it without stopping until it is completed…; likes to complete one task or activity before going onto the next; stays with an activity for a long time; when a toy or game is difficult, quickly turns to another activity) (Fullard et al., 1984) • One item from the Strengths and Difficulties Questionnaire (Sees tasks through to the end, has good attention span) (Goodman, 2001)) • Bottom quintile of scores = ‘low self-regulation’	*Task attentiveness and persistence*	• Independence and Self-regulation domain of the Child Social Behaviour Questionnaire (EPPE) (Likes to work things out for self; Does not need much help with tasks; Chooses activities on own; Persists in the face of difficult tasks; Move to new activity after finishing task) (Johnson et al., 2012) • Bottom quintile of scores = ‘low self-regulation’
**Cognitive ability (6–7 years)**		**Cognitive ability (5 years)**	
*Non-verbal & verbal ability*	• Non-verbal ability: Matrix Reasoning subtest of the Wechsler Intelligence Scale for Children, IV Edition (Wechsler, 2003) • Verbal ability: the Peabody Picture Vocabulary Test (PPVT)-III - LSAC Australian Short-form (Australian Institute of Family Studies, 2011, Rothman, 2005) • Age standardised non-verbal and verbal scores were combined and converted to T-scores, as recommended when using multiple cognitive ability scales (Connelly, 2013) • Bottom quintile of scores = low cognitive ability	*Non-verbal & verbal ability*	• Non-verbal ability: pattern construction subtest of the BAS II(Connelly, 2013) • Verbal ability: BAS II naming vocabulary subtest (Connelly, 2013) • Age standardised non-verbal and verbal scores were combined, converted to T-scores, as recommended when using multiple cognitive ability scales (Connelly, 2013) • Bottom quintile of scores = low cognitive ability
**Baseline confounders (0–1 years)**		**Baseline confounders (9 months)**	
*Maternal young age at first live birth*	Age at first live birth was not directly captured in LSAC, and was estimated using the following: • Mothers' age (years) at the birth of study child • Age (in years) of all other children living in the household • Mother's relationship to these children • Age of mother's eldest biological non-resident child (0−2y, 3−4y, 5−10y, 11−17y, 18y+) • Estimated age at first live birth: <20 years, > = 20 years	*Maternal young age at first live birth*	• Age at first live birth (years): <20 years, > = 20 years
*Language*	• Main language spoken at home by the mother with the study child, coded as ‘English’ or ‘other’	*Language*	• Main household language, coded as ‘English’ or ‘other’
*Ethnicity*^d^	N/A	*Ethnicity*^d^	• White, Black, Indian, Pakistani/Bangladeshi, Mixed, Other
**Intermediate confounding (various ages)**		**Intermediate confounding (various ages)**	
*0–1 years*	• Whether the mother drank alcohol during pregnancy (yes, no)	*9 months*	• Whether the mother drank alcohol during pregnancy (yes, no)
• Whether the mother smoked cigarettes during pregnancy (yes, no)		• Whether the mother smoked cigarettes during pregnancy (yes, no)
*2–3 years*	• Formal childcare use (daycare, preschool or kindergarten)		
*4–5 years*	• Lone parent family • Housing tenure: ‘owned/mortgaged’, ‘renting or other’ • Weekly household income, divided into quintiles • Workless household (no parent in paid employment) • Maternal psychological distress (score>13, Kessler K6 (Kessler et al., 2002)) • Parenting warmth towards the child reported by mother, across six items (Australian Institute of Family Studies, 2011). Scores were highly skewed so lower warmth was defined as < median	*3 years*	• Formal childcare (nursery, childcare centre, or registered childminder) • Lone parent family • Housing tenure: ‘owned/mortgaged’, ‘privately renting’, ‘socially renting or other'^e^ • Equivalised weekly household income (using a modified OECD equivalence scale (Bradshaw and Holmes, 2010)), in quintiles • Workless household (no parent in paid employment) • Maternal psychological distress (score>13, Kessler K6 (Johnson et al., 2012, Kessler et al., 2002)) • Parenting warmth (Pianta scale, ranging from 0 to 35); lower warmth was defined as < 30 (Johnson et al., 2012)

a Year 12: indicates completion of high school.

b GCSE: General Certificate of Secondary Education, received upon completion of high school exams (at approx. age 16 years).

c A-Levels: General Certificate of Education Advanced Level, received upon completion of higher education (approx. 18 years).

d Ethnicity was not collected in LSAC and so was used in a sensitivity analysis of MCS data only.

e The MCS housing tenure variable differentiated between private and social renting, due to large differences in the socio-economic disadvantage and housing needs of these two groups in the UK. The LSAC question on housing tenure does not allow this distinction, the differences between private and social housing are less pronounced in Australia.

**Table 2:** Children with low maths & literacy scores (outcomes), low self-regulation & cognitive ability (mediators), and confounding variables, According to low and high socio-economic disadvantage (SED)^a^: % (N), risk ratios (RR) (95% confidence intervals (CIs)).

Exposure (*X*):	LSAC				MCS			
% (N) low SED	(N) high SED	RR (95% CI), High SED (vs. Low)		% (N) low SED	% (N) high SED	RR (95% CI), High SED (vs. Low)	
**Outcomes (*Y*)**								
Maths score (lowest Q)	16.2 (429)	30.9 (107)	1.91	1.59, 2.28	16.4 (1360)	30.6 (847)	1.87	1.74, 2.01
Literacy score (lowest Q)	16.6 (444)	32.2 (113)	1.94	1.63, 2.30	14.4 (1180)	31.4 (868)	2.19	2.03, 2.36
**Mediators (*M1* and *M2*)**								
Self-regulation score (lowest Q)	21.9 (585)	27.1 (96)	1.24	1.03, 1.49	21.4 (1790)	29.4 (823)	1.37	1.28, 1.47
Cognitive ability (lowest Q)	17.4 (465)	31.1 (110)	1.79	1.50, 2.13	12.8 (1072)	29.8 (834)	2.32	2.14, 2.52
**Baseline confounding (*C*)**								
<20 years at 1st birth	1.9 (51)	15.8 (56)	8.29	5.77, 11.92	11.2 (933)	35.0 (979)	3.13	2.90, 3.39
Non-English language	11.5 (308)	9.3 (33)	0.81	0.58, 1.14	8.0 (671)	16.2 (453)	2.01	1.80, 2.25
**Intermediate confounding (*L*)**								
Less supportive environment	8.7 (232)	21.9 (75)	2.44	1.93, 3.09	15.6 (1306)	48.4 (1355)	3.10	2.90, 3.30

LSAC: Longitudinal Study of Australian Children; MCS: Millennium Cohort Study; Q: quintile;.

a High SED is defined as not completing year 12 (LSAC) or not achieving GCSE grades A*-C (MCS); *N* = *3028 in LSAC; 11,168 in MCS (complete variable set for those with maths or literacy scores)*.

**Table 3:** % (N) of children in the lowest quintile of maths and literacy scores (outcomes), in children who were and were not in the lowest quintile of self-regulation and cognitive ability (mediators).

	LSAC		MCS	
Maths scores % (N) lowest Q	Literacy scores % (N) lowest Q	Maths scores % (N) lowest Q	Literacy scores % (N) lowest Q
**Self-regulation (M1)**				
Lowest Q	28.2 (190)	30.8 (209)	27.9 (723)	25.9 (664)
Other Qs	15.0 (346)	14.9 (348)	17.4 (1484)	16.4 (1384)
**Cognitive ability (M2)**				
Lowest Q	36.1 (205)	36.0 (206)	47.4 (893)	41.1 (770)
Other Qs	13.7 (331)	14.3 (351)	14.3 (1314)	14.0 (1278)

LSAC: N = 3028.

MCS: N = 11,168.

Q: quintile; LSAC = Longitudinal Study of Australian Children; MCS = Millennium Cohort Study.

**Table 4:** Risk Ratios (RRs) and 95% CIs for the Direct and Indirect pathways From Low Socio-economic Disadvantage (SED^e^) to Low Maths and Literacy Scores, Before (Section A) and After (Sections B and C) Adjustment for Intermediate Confounding^d^.

	LSAC		MCS	
RRs	95% CI	RRs	95% CI
**Pathways from SED to low Maths scores**				
*Section A: Self-regulation and cognitive ability*				
Direct^a^	1.46	1.17, 1.79	1.46	1.34, 1.58
Joint indirect^a^	1.19	1.10, 1.32	1.18	1.14, 1.22
Indirect via self-regulation^b^	1.05	1.01, 1.11	1.02	1.01, 1.03
Indirect via cognitive ability^b^	1.13	1.06, 1.22	1.16	1.12, 1.19
*Section B: Self-regulation, adj. intermediate confounding (L*^d^*)*				
Indirect via self-regulation, adj. L^d^^,^^c^	1.05	1.01, 1.11	1.02	1.01, 1.03
*Section C: Cognitive ability, adj. intermediate confounding (L*^d^*)*				
Indirect via cognitive ability, adj. L^d^^,^^c^	1.14	1.07, 1.25	1.16	1.13, 1.20
**Pathways from SED to low Literacy scores**				
*Section A: Self-regulation and cognitive ability*				
Direct^a^	1.51	1.22, 1.86	1.75	1.61, 1.90
Joint indirect^a^	1.16	1.08, 1.28	1.16	1.12, 1.19
Indirect via self-regulation^b^	1.05	1.01, 1.10	1.02	1.01, 1.04
Indirect via cognitive ability^b^	1.11	1.05, 1.20	1.12	1.09, 1.15
*Section B: Self-regulation, adj. intermediate confounding (L*^d^*)*				
Indirect via self-regulation, adj. L^d^^,^^c^	1.04	1.01, 1.11	1.02	1.01, 1.04
*Section C: Cognitive ability, adj. intermediate confounding (L*^d^*)*				
Indirect via cognitive ability, adj. L^d^^,^^c^	1.13	1.06, 1.23	1.12	1.09, 1.15

LSAC = Longitudinal Study of Australian Children; MCS = Millennium Cohort Study.

*N* = 3028 (LSAC) and 11,168 (MCS).

All analyses adjust for baseline confounding: Young age (<20) at first live birth; English language spoken in the home.

a Estimated using method 1 ‘joint effects’.

b Estimated using method 2 ‘path specific effects’.

c Estimated using method 3 ‘intervention analogue’.

d Latent class measure representing the ‘Early home and parenting environment’.

e Low maternal education (left high school without Year 12 qualifications (Australia) or GCSEs grades A*-C (UK)).

**Table 5:** Risk differences (RDs) and 95% CIs for the direct and indirect pathways from socio-economic disadvantage (SED^e^) to low maths and literacy scores, before (Section A) and after (Sections B and C) adjustment for intermediate confounding^d^.

	LSAC		MCS	
RDs	95% CI	RDs	95% CI
**Pathways from SED to low Maths scores**				
*Section A: Self-regulation and cognitive ability*				
Direct^a^	7.51%	2.87, 12.59	7.85%	5.93, 9.72
Joint indirect^a^	4.45%	2.45, 7.12	4.52%	3.68, 5.45
Indirect via self-regulation^b^	1.4%	0.38, 2.95	0.62%	0.31, 1.00
Indirect via cognitive ability^b^	3.00%	1.37, 5.10	3.88%	3.13, 4.73
*Section B: Self-regulation, adj. intermediate confounding (L*^d^*)*				
Indirect via self-regulation, adj. L^d^^,^^c^	1.12%	−0.13, 1.63	0.59%	0.28, 0.96
*Section C: Cognitive ability, adj. intermediate confounding (L*^d^*)*				
Indirect via cognitive ability, adj. L^d^^,^^c^	3.45%	1.73, 6.05	4.1%	3.34, 4.99
**Pathways from SED to low Literacy**				
*Section A: Self-regulation and cognitive*				
Direct^a^	8.71%	3.87, 14.06	11.52%	9.57, 13.48
Joint indirect^a^	4.13%	2.09, 6.69	3.96%	3.19, 4.84
Indirect via self-regulation^b^	1.32%	−0.03, 2.80	0.75%	0.42, 1.14
Indirect via cognitive ability^b^	2.76% 0.60	1.20, 4.77 (−0.04, 1.79)	3.21%	2.52, 4.01
*Section B: Self-regulation, adj. intermediate confounding (L*^d^*)*				
Indirect via self-regulation, adj. L^d^^,^^c^	1.18%	−0.23, 2.70	0.70%	0.38, 1.08
*Section C: Cognitive ability, adj. intermediate confounding (L*^d^*)*				
Indirect via cognitive ability, adj. L^d^^,^^c^	3.45%	1.61, 5.76	3.31%	2.60, 4.12

LSAC = Longitudinal Study of Australian Children; MCS = Millennium Cohort Study.

*N* = 3028 (LSAC) and 11,168 (MCS).

All analyses adjust for baseline confounding: Young age (<20) at first live birth; English language spoken in the home.

a Estimated using method 1 ‘joint effects’.

b Estimated using method 2 ‘path specific effects’.

c Estimated using method 3 ‘intervention analogue’.

d Latent class measure representing the ‘Early home and parenting environment’.

e Low maternal education (left high school without Year 12 qualifications (Australia) or GCSEs grades A*-C (UK)).
