# Supplementary material for: Do early life cognitive ability and self-regulation skills explain socio-economic inequalities in academic achievement? An effect decomposition analysis in UK and Australian cohorts
Source: Soc Sci Med. 2016 Sep;165:108–18. doi: 10.1016/j.socscimed.2016.07.016 (PMC5012893; doi:10.1016/j.socscimed.2016.07.016)
Supplement: Supplementary file 1 [file mmc1.docx]

**Appendix A. Additional information on the latent class analysis used to create the binary measure of intermediate confounding**

It was only possible to adjust for a single, binary intermediate confounder in the analysis. Therefore we used latent class analysis (LCA) to represent confounding based on the following factors: alcohol and smoking in pregnancy, lone parenthood, maternal psychological distress, tenure, household income, parenting style, workless households and formal childcare attendance. Two sets of parameters were estimated: class membership probabilities (reflecting the relative size of the two classes) and item response probabilities (the probability of members in each class displaying each of the confounding factors). Children were assigned to the class that they had the highest probability of belonging to(Lanza, Dziak, Huang, Wagner, & Collins, 2014). As shown in Table A1, the two class measure had higher entropy and class posterior probabilities in both cohorts (as compared to the three class measure), although the Bayesian Information Criterion (BIC), was larger.

*Table A1: factors used to assess the latent class models (for two to three classes)*

|  | **LSAC** | | | **MCS** | | |
| --- | --- | --- | --- | --- | --- | --- |
|  | **1 class** | **2 class** | **3 class** | **1 class** | **2 class** | **3 class** |
| *Posterior probabilities:* | | | |  | | |
|  | 1 | 0.98 | 0.80 | 1 | 0.96 | 0.86 |
|  |  | 0.92 | 0.73 |  | 0.91 | 0.95 |
|  |  |  | 0.78 |  |  | 0.86 |
| *Entropy:* | | | |  | | |
|  | 1 | 0.89 | 0.59 | 1 | 0.82 | 0.72 |
| *Bayesian Information Criterion (BIC):* | | | |  | | |
|  | 2280 | 988 | 910 | 15159 | 3883 | 2261 |

Posterior probabilities: 1 indicates perfect assignment within that class; Entropy: 1 indicates perfect assignment of all individuals to all classes; Bayesian Information Criterion (BIC): lower values indicate a more parsimonious model

**References**

1. Lanza S, Dziak J, Huang L, Wagner A, Collins L. LCA Stata Plugin Users' Guide: Version 1.1. Penn State: University Park: The Methodology Center, 2014.
