# Supplementary material for: Do early life cognitive ability and self-regulation skills explain socio-economic inequalities in academic achievement? An effect decomposition analysis in UK and Australian cohorts
Source: Soc Sci Med. 2016 Sep;165:108–18. doi: 10.1016/j.socscimed.2016.07.016 (PMC5012893; doi:10.1016/j.socscimed.2016.07.016)
Supplement: Supplementary file 2 [file mmc2.docx]

**Appendix B. Technical detail and Stata commands for the three analytical approaches used to estimate indirect and direct pathways (Vanderweele, Vansteelandt, & Robins, 2014)**

Method one: ‘Joint Indirect Effects’

The direct pathway (i.e. the association between SED and academic achievement not occurring through self-regulation or cognitive ability) is given by the following.

1. E$\left( Y_{{aM1}_{a^{*}}{M2}_{a^{*}}}- Y_{{a^{*}M1}_{a^{*}}{M2}_{a^{*}}} \right)|C$

Where the exposure takes values *a*=1 or *a**=0, *M1* and *M2* are the mediators (where *M1* is a cause of *M2*)*; M1_a*_* is the value that *M1* takes when *a**=0 and *M1_a_* is the value that *M1* takes when *a*=1, and so forth). *Y* is the outcome and *C* represents baseline confounding.

The indirect pathway (i.e. the association between the exposure and outcome that is occurring through either mediator) is estimated by the change in the risk of the outcome when the mediators change from *M1_a_* to *M1_a*_* and from *M2_a_* to *M2_a*_*, while the exposure is held at *a*:

1. E$\left( Y_{{aM1}_{a}{M2}_{a}}- Y_{{aM1}_{a^{*}}{M2}_{a^{*}}} \right)|C$

The data are duplicated twice and a new variable, *a**, is assigned a value of 0 in the first duplication and 1 in the second. Each line of data is assigned an inverse probability of treatment weight (IPTW):

1. P$\left( M1 | a^{*}, C \right)$ P$\left( M2 | a, C \right)$ / P$\left( X | C \right)$ P$\left( M1 | a, C \right)$ P$\left( M2 | M1,a, C \right)$

The direct pathway is obtained from the coefficient of *X* in the weighted regression of the outcome on the exposure, in cases where *a**=0, and the indirect pathway from the coefficient of *a** when *a*=1.

Method two: ‘Path Specific Effects’

Technique two enables decomposition of the indirect pathways from the exposure to the outcomes that act via the two mediators separately. The indirect pathway through cognitive ability (*M2*) only is given by the average difference in outcome when *M2* is changed from *M2_a_* to *M2_a*_*; while the exposure is held at *a* and *M1* is held at *M1_a_*_*_.

1. E$\left( Y_{{aM1}_{a^{*}}{M2}_{{aM1_{a^{*}}}}}- Y_{{aM1}_{a^{*}}{M2}_{a^{*}}} \right)|C$

The indirect pathway through *M1* is given by the average difference in the outcome when only *M1* is changed from *M1_a_* to *M1_a_*_*_. The exposure is held at *a*. Because *M2* is caused by both the exposure and *M1*, it is held at *M2_aM1a*_.*

1. E$\left( Y_{a{M2}_{a}}- Y_{{aM1}_{a^{*}}{M2}_{aM1_{a^{*}}}} \right)|C$

The direct pathway is given by the same formula used in approach 1 (formula i).

Three copies of the data are created, with two new variables a* and a**; a* takes on the same value as observed a in the first copy and the counterfactual in the second and third; and a** takes on observed a in the first two copies and the counterfactual in the third. The expected values of *M1* and *M2* under counterfactual a were estimated with the IPTW:

1. P$\left( M1 | a^{*}, C \right)$ P$\left( M2 | {M1, a}^{**}, C \right)$ / P$\left( a | C \right)$ P$\left( M1 | a, C \right)$ P$\left( M2 | M1,a, C \right)$

The direct pathway is given by the coefficient of a in the weighted regression when a *= a**=0. The indirect pathway through *M1* is given by the coefficient of a* when a= a**=1; and the indirect pathway through *M2* is given by a** when a=1; a*=0.

Approach three: Intervention Analogue Effects

In approach three the value of *M2* is estimated as if it had been assigned independently of *M1*, using the following weight:

1. $\sum_{M1} P\left( M1 | a^{*}, C \right)$ *P*$\left( M2 | {M1, a}^{*}, C \right)$ / *P*$\left( a | C \right)$ *P*$\left( M2 | M1,a, C \right)$

The indirect pathway from the exposure to the outcome via *M2* is given by the average change in the outcome had the value of *M2* been randomized within the distribution of a* as opposed to a; the exposure is held at a:

1. E$\left( Y_{aG_{a}} \right)$ - E$\left( Y_{aG_{a*}} \right)$

Where *G_a_* denotes the random draw of *M2* for children with a*,* and *G_a*_* for children with a***.

The direct pathway is given by the average difference in the outcome when the exposure changes from a to a***, and *M2* is held at the value it would have been randomly assigned within the distribution of a***:

1. E$\left( Y_{G_{a*}} \right)$ - E$\left( Y_{a*G_{a*}} \right)$

(where *G_a_* denotes the random draw of *M2* for children with a*,* and *G_a*_* for children with a***).

The data are replicated and the estimates derived in the same way as in technique 1.

The direct pathway is obtained from the coefficient of a in the weighted regression of the outcome on the exposure in cases where a*=0. The indirect pathway through *M2* is obtained from the coefficient of a* when a=1.

Stata code:

*********************************

*Method 1: joint mediators

*********************************

use /*"enter dataset name here"*/, clear

capture program drop myboot2

program define myboot2,rclass

preserve

bsample

drop /*enter dataset id here*/

gen id=_n

*#1 create additional lines for counterfactual data

expand 2,gen(dataset)

sort id dataset

gen astar1=0 if dataset==0

replace astar1=1 if dataset==1

*#2 create inverse probability treatment weight

*weights for M

gen expo_temp=a

mlogit m i.expo_temp L c1 c2 if dataset==0

predict out0 out1, p

gen denomM=out0 if m==0

replace denomM =out1 if m==1

drop out0 out1

replace expo_temp=astar

predict out0 out1, p

gen numM=out0 if m==0

replace numM =out1 if m==1

drop out0 out1

*weights for L

replace expo_temp=a

mlogit L i.expo_temp c1 c2 if dataset==0

predict out0 out1, p

gen denomL=out0 if L==0

replace denomL =out1 if L==1

drop out0 out1

replace expo_temp=astar

predict out0 out1, p

gen numL=out0 if L==0

replace numL =out1 if L==1

drop out0 out1

*weight for A

mlogit a c1 c2 if dataset==0

predict out0 out1, p

gen denomX=out0 if x==0

replace denomA =out1 if a==1

drop out0 out1

gen int denomM2=denomM

gen num=(numM * numL)

gen denom= (denomM*denomL*denomA)

gen weight=(num/denom)

*#3 estimated coefficients

logistic y a if astar==0 [pweight=weight] ,vce(cluster id) or

matrix bEst=e(b)

return scalar Method1_DE = bEst[1,1]

logistic y astar if a==1 [pweight=weight] ,vce(cluster id) or

matrix bEst=e(b)

return scalar Method1_IE = bEst[1,1]

/*Store estimation results*/

restore

end

*#4 estimated confidence intervals

/*use bootstrap program*/

simulate Method1_DE=r(Method1_DE) Method1_IE=r(Method1_IE), reps(5000) nodots seed (12345):myboot2

matrix limits = J(2,3,0)

centile Method1_DE`j', centile(2.5 50 97.5)

scalar Method1_DE_LL`j' = r(c_1)

scalar Method1_DE`j' = r(c_2)

scalar Method1_DE_UL`j' = r(c_3)

matrix limits [1,1] = exp(Method1_DE)

matrix limits [1,2] = exp(Method1_DE_LL)

matrix limits [1,3] = exp(Method1_DE_UL)

centile Method1_IE`j', centile(2.5 50 97.5)

scalar Method1_IE_LL`j' = r(c_1)

scalar Method1_IE`j' = r(c_2)

scalar Method1_IE_UL`j' = r(c_3)

matrix limits [2,1] = exp(Method1_IE)

matrix limits [2,2] = exp(Method1_IE_LL)

matrix limits [2,3] = exp(Method1_IE_UL)

matrix colnames limits = Coeff LL UL

matrix rownames limits = Method1_DE Method1_IE

matlist limits

svmat limits, name(Coeff)

keep Coeff*

rename Coeff2 LL

rename Coeff3 UL

gen idcleanup=_n

drop if idcleanup>2

gen str15 varlabel = " "

replace varlabel = "Method1_DE" if idcleanup==1

replace varlabel = "Method1_IE" if idcleanup==2

drop idcleanup

save /*enter new dataset name here*/, replace

*********************************

*Method 2: path specific

*********************************

use "mydata", clear

capture program drop myboot2

program define myboot2,rclass

preserve

bsample

drop /*datasetid*/

gen id=_n

*#1 create additional lines for counterfactual data

expand 3,gen(dataset)

sort id dataset

replace dataset=dataset[_n-1]+1 if dataset==1

gen astar1=aa if dataset==0

replace astar1=(1-a) if dataset==1 |dataset==2

gen astar2=aa if dataset==0 | dataset==1

replace astar2=(1-a) if dataset==2

*#2 create inverse probability treatment weight

*weights for M1

gen expo_temp=a

mlogit m i.expo_temp L c1 c2 if dataset==0

predict out0 out1, p

gen denomM=out0 if m==0

replace denomM =out1 if m==1

drop out0 out1

replace expo_temp=astar2

predict out0 out1, p

gen numM=out0 if m==0

replace numM =out1 if m==1

drop out0 out1

*weights for L

replace expo_temp=a

mlogit L i.expo_temp c1 c2 if dataset==0

predict out0 out1, p

gen denomL=out0 if L==0

replace denomL =out1 if L==1

drop out0 out1

replace expo_temp=astar1

predict out0 out1, p

gen numL=out0 if L==0

replace numL =out1 if L==1

drop out0 out1

*weight for A

mlogit a c1 c2 if dataset==0

predict out0 out1, p

gen denomA=out0 if a==0

replace denomA =out1 if a==1

drop out0 out1

gen num=(numM * numL)

gen denom= (denomM*denomL*denomA)

gen weight=(num/denom)

*#3 estimated coefficients

logistic y a if astar1==0 & astar2==0 [pweight=weight] ,vce(cluster id) or

matrix bEst=e(b)

return scalar Method2_DE = bEst[1,1]

logistic y astar2 if a==1 &astar1==0 [pweight=weight] ,vce(cluster id) or

matrix bEst=e(b)

return scalar Method2_IME = bEst[1,1]

logistic y astar1 if a==1 &astar2==1 [pweight=weight] ,vce(cluster id) or

matrix bEst=e(b)

return scalar Method2_ILE = bEst[1,1]

/*Store estimation results*/

restore

end

*#4 estimated confidence intervals

/*use bootstrap program*/

simulate Method2_DE Method2_IME Method2_ILE, reps(5000) seed (12345):myboot2

centile Method2_DE`j', centile(2.5 50 97.5)

scalar Method2_DE_LL`j' = r(c_1)

scalar Method2_DE`j' = r(c_2)

scalar Method2_DE_UL`j' = r(c_3)

matrix limits = J(3,3,0)

matrix limits [1,1] = exp(Method2_DE)

matrix limits [1,2] = exp(Method2_DE_LL)

matrix limits [1,3] = exp(Method2_DE_UL)

centile Method2_IME`j', centile(2.5 50 97.5)

scalar Method2_IME_LL`j' = r(c_1)

scalar Method2_IME`j' = r(c_2)

scalar Method2_IME_UL`j' = r(c_3)

matrix limits [2,1] = exp(Method2_IME)

matrix limits [2,2] = exp(Method2_IME_LL)

matrix limits [2,3] = exp(Method2_IME_UL)

centile Method2_ILE`j', centile(2.5 50 97.5)

scalar Method2_ILE_LL`j' = r(c_1)

scalar Method2_ILE`j' = r(c_2)

scalar Method2_ILE_UL`j' = r(c_3)

matrix limits [3,1] = exp(Method2_ILE)

matrix limits [3,2] = exp(Method2_ILE_LL)

matrix limits [3,3] = exp(Method2_ILE_UL)

matrix colnames limits = Coeff LL UL

matrix rownames limits = Method2_DE Method2_IME Method2_ILE

matlist limits

svmat limits, name(Coeff)

keep Coeff*

rename Coeff2 LL

rename Coeff3 UL

gen idcleanup=_n

drop if idcleanup>3

gen str15 varlabel = " "

replace varlabel = "Method2_DE" if idcleanup==1

replace varlabel = "Method2_IME" if idcleanup==2

replace varlabel = "Method2_ILE" if idcleanup==3

drop idcleanup

save /*enter new dataset name here*/, replace

*********************************

*Method 3: intervention analogue

*********************************

use "mydata", clear

capture program drop myboot2

program define myboot2,rclass

preserve

bsample

drop /*datasetid*/

gen id=_n

*#1 create additional lines for counterfactual data

expand 2,gen(dataset)

sort id dataset

gen astar1=0 if dataset==0

replace astar1=1 if dataset==1

*#2 create inverse probability treatment weight

*weights for M1 - denom

gen expo_temp=a

mlogit m i.expo_temp L c1 c2 if dataset==0

predict out0 out1, p

gen denomM=out0 if m==0

replace denomM =out1 if m==1

drop out0 out1

*weights for m, num separate for levels of L

replace expo_temp=a

mlogit m i.expo_temp L c1 c2 if dataset==0 & L==0

replace expo_temp=astar

predict out0 out1, p

gen numM0=out0 if m==0

replace numM0 =out1 if m==1

drop out0 out1

replace expo_temp=a

mlogit m i.expo_temp L c1 c2 if dataset==0 & L==1

replace expo_temp=astar

predict out0 out1, p

gen numM1=out0 if m==0

replace numM1 =out1 if m==1

drop out0 out1

*weights for L

replace expo_temp=a

mlogit L i.expo_temp c1 c2 if dataset==0

replace expo_temp=astar

predict out0 out1, p

gen numL0=out0

gen numL1=out1

drop out0 out1

*weight for A

mlogit a c1 c2 if dataset==0

predict out0 out1, p

gen denomA=out0 if a==0

replace denomA =out1 if a==1

drop out0 out1

*****numerator needs to be multipled within levels of L, and then added up

gen numlevel1= numM1*numL1

gen numlevel0= numM0*numL0

gen numlevels=numlevel1+numlevel0

gen denom= (denomM*denomA)

gen weight=(numlevels/denom)

*#3 estimated coefficients

logistic y a if astar==0 [pweight=weight] ,vce(cluster id) or

matrix bEst=e(b)

return scalar Method3_DE = bEst[1,1]

logistic y astar if a==1 [pweight=weight] ,vce(cluster id) or

matrix bEst=e(b)

return scalar Method3_IE = bEst[1,1]

/*Store estimation results*/

restore

end

*#4 estimated confidence intervals

/*use bootstrap program*/

simulate Method3_DE=r(Method3_DE) Method3_IE=r(Method3_IE), reps(5000) nodots seed (12345):myboot2

matrix limits = J(2,3,0)

centile Method3_DE`j', centile(2.5 50 97.5)

scalar Method3_DE_LL`j' = r(c_1)

scalar Method3_DE`j' = r(c_2)

scalar Method3_DE_UL`j' = r(c_3)

matrix limits [1,1] = exp(Method3_DE)

matrix limits [1,2] = exp(Method3_DE_LL)

matrix limits [1,3] = exp(Method3_DE_UL)

centile Method3_IE`j', centile(2.5 50 97.5)

scalar Method3_IE_LL`j' = r(c_1)

scalar Method3_IE`j' = r(c_2)

scalar Method3_IE_UL`j' = r(c_3)

matrix limits [2,1] = exp(Method3_IE)

matrix limits [2,2] = exp(Method3_IE_LL)

matrix limits [2,3] = exp(Method3_IE_UL)

matrix colnames limits = Coeff LL UL

matrix rownames limits = Method3_DE Method3_IE

matlist limits

svmat limits, name(Coeff)

keep Coeff*

rename Coeff2 LL

rename Coeff3 UL

gen idcleanup=_n

drop if idcleanup>2

gen str15 varlabel = " "

replace varlabel = "Method3_DE" if idcleanup==1

replace varlabel = "Method3_IE" if idcleanup==2

drop idcleanup

save /*enter new dataset name here*/, replace

***********************************

*Sensitivity analysis to unmeasured confounding

***********************************

use "data", clear

***obtain mean values of baseline confounders

sum c1

sca c1m=r(mean)

sum c2

sca c2m=r(mean)

***estimate probability of outcome if exposure = 0

logit y1 c1 c2 if a==0

gen py_A0=exp(_cons+ _b[c1] * c1m + _b[c2] * c2m)

sum py_A0

sca pya0=r(mean)

***estimate probability of outcome if exposure = 1

logit y1 c1 c2 if a==1

gen py_A1=exp(_cons+ _b[c1] * c1m + _b[c2] * c2m)

sum py_A1

sca pya1=r(mean)

***estimate probability of mediator

logit m1 c1 c2 if a==0

gen pm=exp(_cons+ _b[c1] * c1m + _b[c2] * c2m)

***create variables representing degree of suspected bias

sca g1_c=.05

sca g0_c= 0.025

sca G_c=g1_c*pm+g0_c*(1-pm)

sca pm=r(mean)

gen Bdc=G_c/pya0

gen Bic=G_c/pya1

sum Bdc

sca bdc=r(mean)

sum Bic

sca bic=r(mean)

***rescale original results to account for bias

sca IE_corrected = /*ORIG*/ 2.50/(1-(/*ORIG*/*bic))

sca DE_corrected=/*ORIG*/-bdc

sca list

Vanderweele, T. J., Vansteelandt, S., & Robins, J. M. (2014). Effect decomposition in the presence of an exposure-induced mediator-outcome confounder. *Epidemiology, 25*(2), 300-306. doi:10.1097/ede.0000000000000034
