# Supplementary material for: Do early life cognitive ability and self-regulation skills explain socio-economic inequalities in academic achievement? An effect decomposition analysis in UK and Australian cohorts
Source: Soc Sci Med. 2016 Sep;165:108–18. doi: 10.1016/j.socscimed.2016.07.016 (PMC5012893; doi:10.1016/j.socscimed.2016.07.016)
Supplement: Supplementary file 3 [file mmc3.docx]

**Appendix C. Characteristics of those with and without missing data**

*Table A2: prevalence of outcome, exposure, mediator, and confounder variables in those with complete data (on relevant variable) and complete cases*

|  | **LSAC** | | **MCS** | |
| --- | --- | --- | --- | --- |
|  | Whole sample  *N*=5107 | Complete case (with MAR for *L*)  *N*=3028 | Whole sample  *N=*18,296 | Complete case (with MAR for  *L*)  *N=*11,168 |
| *Outcomes* | | | | |
| Math’s score: lowest quintile (%, N) | 19.9 (680) | 17.9 (536) | 22.1 (2942) | 19.9 (2207) |
| Literacy score: lowest quintile (%, N) | 20.3 (702) | 18.4 (557) | 20.2 (2674) | 18.6 (2048) |
| *Exposure* | | | | |
| Low education | 16.8 (847) | 11.7 (354) | 33.7 (6367) | 25.1 (2802) |
| *Mediators* |  |  |  |  |
| Cognitive ability: bottom quintile (%, N) | 21.3 (889) | 19.0 (575) | 21.5 (3160) | 17.1 (1906) |
| Self-regulation: bottom quintile (%, N) | 23.9 (1005) | 22.5 (681) | 24.3 (3371) | 23.4 (2661) |
| *Baseline confounder variables* | | | | |
| Young age (< 20 years) at 1^st^ live birth | 5.4 (251) | 3.5 (107) | 21.0 (3706) | 17.1 (1912) |
| Non-English language @ home (%, N) | 14.5 (740) | 11.3 (341) | 15.1 (2769) | 10.1 (1124) |

L: intermediate confounding represented by a latent variable which was created under a Missing at Random Assumption (see Online Resource B). Missing data, LSAC: math score 1685, literacy score 1652, maternal education 62, cognitive ability 935, self-regulation 898, maternal age at first live birth 452, main language spoken with child 3.

Missing data, MCS: math score 5663, reading score 5740, maternal education 88, cognitive ability 4282, self-regulation 5129, maternal age at first live birth 1359, main language spoken in home 685.
