# Supplementary material for: Do early life cognitive ability and self-regulation skills explain socio-economic inequalities in academic achievement? An effect decomposition analysis in UK and Australian cohorts
Source: Soc Sci Med. 2016 Sep;165:108–18. doi: 10.1016/j.socscimed.2016.07.016 (PMC5012893; doi:10.1016/j.socscimed.2016.07.016)
Supplement: Supplementary file 4 [file mmc4.docx]

**Appendix D. Sensitivity analysis carried out in the ALSPAC cohort with more objective measures of self-regulation (and cognitive ability)**

The aim of this sensitivity analyses was to consider whether the indirect effects of self-regulation were underestimated in the Millennium Cohort Study (MCS) and Longitudinal Study of Australian Children (LSAC) analyses, because they were based on parental report. Using data from the UK Avon Longitudinal Study of Parents and Children (ALSPAC)^5 6^, we assessed the effects of maternal education (captured in pregnancy) on young people’s GCSE grades (at 15.5 years) via self-regulation (at 10.5 years) and cognitive ability (at 15.5 years). Please note that the study website contains details of all the data that is available through a fully searchable data dictionary (<http://www.bristol.ac.uk/alspac/researchers/access/>). Ethical approval for the study was obtained from the ALSPAC Ethics and Law Committee and the Local Research Ethnics Committees. Cognitive ability was represented by a measure of Intelligence captured using the Wechsler Abbreviated Intelligence Scale (bottom quintile). Self-regulation was represented using a measure of executive function, assessed using a battery of items from the Test of Everyday Attention in Children, the Stop Signal Task, and the Counting Span Task for working memory (bottom quintile). These items included additional traits (such as inhibitory control and working memory) not included in the LSAC and MCS measures (which focused on task attainment and persistence); however to include a more encapsulating measure of children’s “non-cognitive” abilities was appropriate, since the aim was to investigate whether the indirect effect via self-regulation in the main analyses had been underestimated. As seen in the LSAC and MCS analyses, around two thirds of the total effect of maternal education on children’s exam performance was direct, and the majority of the indirect effect was through cognitive ability and not self-regulation.

*Table A3: Risk ratios (RRs) for poor GCSE* achievement, according to socio-economic disadvantage (SED**): direct and indirect pathways via self-regulation and cognitive ability across early-late adolescence: Avon Longitudinal Study of Parents and their Children (N=3102)*

|  | **RR (95% CIs)** |
| --- | --- |
| ***Section A:*** *Self-regulation (M1) and cognitive ability (M2)* | |
| Direct^1^ | 1.52 (1.34, 1.70) |
| Joint indirect ^1^ | 1.22 (1.16, 1.30) |
| Indirect via self-regulation^2^ | 1.02 (1.00, 1.04) |
| Indirect via cognitive ability^2^ | 1.20 (1.14, 1.28) |
| ***Section B:*** *Self-regulation (M1) and intermediate confounding (L*^*)* | |
| Direct pathway, adj. *L*^3^  Indirect via self-regulation, adj. *L*^3^ | 1.83 (1.64, 2.03) |
|  | 1.02 (1.00, 1.04) |
| ***Section C:*** *Cognitive ability (M2) and intermediate confounding (L*^*)* | |
| Direct pathway, adj. *L*^3^  Indirect via cognitive ability, adj. *L*^3^ | 1.55 (1.37, 1.74) |
|  | 1.20 (1.14, 1.28) |

*did not achieve at least five grades A*-C (including math and English) on their General Certificate in Secondary Education (GCSE) exams

**low maternal education (< degree)

^1^Estimated using method 1 ‘joint effects’; ^2^Estimated using method 2 ‘path specific effects’; ^3^Estimated using method 3 ‘intervention analogue’

^latent class measure representing the ‘Early home and parenting environment’: smoking regularly since birth, income, marital status, maternal alcohol use, postnatal depression, breastfeeding duration, attachment, number of books in the home.

**References**

Johnson, J., Schoon, I., Joshi, H., & Smith, K. (2012). Millennium Cohort Study: Psychological, Developmental and Health Inventories. London: Centre for Longitudinal Studies.

Lanza, S., Dziak, J., Huang, L., Wagner, A., & Collins, L. (2014). LCA Stata Plugin Users' Guide: Version 1.1. Penn State: University Park: The Methodology Center.
